# Immunohistochemical expression of bcl-2; an apoptosis regulatory protein in squamous cell carcinoma of oropharynx: A diagnostic cross-sectional study

**DOI:** 10.1016/j.amsu.2021.102480

**Published:** 2021-06-11

**Authors:** Namita Bhutani, Pooja Poswal, Shilpi Moga, Sunil Arora

**Affiliations:** a Deptt. of Pathology, North DMC Medical College and Hindu Rao Hospital, India; b Deptt. of Pathology, SGT Medical College & University, Gurugram, Haryana, India

**Keywords:** Apoptotic index, Bcl-2, Human papilloma virus, Immunohistochemistry, Oropharyngeal squamous cell carcinoma

## Abstract

**Background:**

The Oropharyngeal squamous cell carcinoma is the second most common head and neck malignancy. Bcl-2 expression alterations have been reported invariably in different cancers. It plays a part in carcinogenesis by inhibiting programmed cell death and thus increasing cell survival.

**Materials and methods:**

The present study was conducted in Department of Pathology, S.G.T. Medical College and University, Gurugram over a period of one year (2019–20) on biopsy proven cases of squmaous cell carcinoma of oropharynx. Grading of the tumor was done using Anneroth's multifactorial grading system. The Bcl-2 scoring was done.

**Results:**

In the present study, a total of 75 cases of oropharyngeal SCC constituted the study group, with the mean age at presentation of 56.63 years. The correlation between Anneroth's grading system and WHO grade was found to be statistically significant, while correlation between WHO grade with lymph node status was found to be statistically non significant.

**Conclusion:**

There was significant correlation between Anneroth's grading system and WHO grading system in SCC of oropharynx and it was found to be more relevant in predicting the stage of tumor. Bcl-2 expression did not correlate with the grading of tumor.

## Highlights

• The burden of head and neck cancer in India can be regarded as ‘tip of iceberg’ situation’.
• Bcl-2 plays a part in carcinogenesis by inhibiting programmed cell death and thus increasing cell survival.
• The expression of bcl-2 rises with increasing grade of tumor.
• Bcl-2 status is closely associated to the success of chemoradiation treatment of oropharyngeal SCC.

## 1 Introduction

The Oropharyngeal squamous cell carcinoma (SCC) is the second most frequent head and neck malignancy accounting for 90% of the cases. The actual burden of head and neck cancer in India is much greater than reflected through the existing literature and hence can be regarded as ‘tip of iceberg’ situation’ [1]. These cancers are more commonly seen in males in their sixth or seventh decades of life [2,3]. The risk factors include tobacco smoke, moderate to heavy alcohol consumption, human papillomavirus (HPV) and positive family history. The incidence of oropharyngeal cancer is increasing among younger age groups, and this may be atributed to an increased association with HPV [3]. A number of oral premalignant lesions have been identified, including leukoplakia, erythroplakia, mixed red and white lesions, lichen planus and verrucous lesions. The potential for malignant transformation of these lesions appear to correlate with the degree of dysplasia it exhibits [4]. (see Fig. 8, Fig. 9, Fig. 10, Fig. 11, Fig. 12)

Various protooncogenes including bcl-2 have been categorised as genes regulating the programmed cell death (apoptosis). Bcl-2 contributes to neoplastic cell growth not by accelerating rate of cellular proliferation but by prolonging the cell survival through inhibition of programmed cell death [5]. Bcl-2 expression alterations have been reported invariably in different cancers. There are variable and inconsistent reports in literature regarding expression of bcl-2 in squamous cell carcinoma of oropharynx by various authors. Bcl-2 is reported as a possible prognostic marker and factor altering sensitivity of tumor to radiotherapy. The Aim of our study was to study the bcl-2 expression in squamous cell carcinoma of oropharynx and the main objectives of the study were to grade squamous cell carcinoma of oropharynx using Anneroth's multifactorial grading system, to study the bcl-2 expression in various grades of squamous cell carcinoma in the study group and to compare the results of bcl-2 expression in various grades of squamous cell carcinoma of oropharynx.

## 2 Materials and methods

The present study was conducted in Department of Pathology, S.G.T. Medical College and University, Gurugram over a period of one year (2019–20) on biopsy proven cases of squamous cell carcinoma of oropharynx. A total of 75 cases were included in the study. After obtaining the Ethical clearance from the Institutional Review Board, vide no. FMHS/MD/23112020 (Certificate attached), The tissue biopsies were processed and wax blocks prepared. They were subjected to routine haematoxylin and eosin stain [6]. Patients with carcinomas other than squamous cell carcinoma and who received prior chemo/radiotherapy were excluded. Grading of the tumor was done using Anneroth's multifactorial grading system [7]. This system consists of evaluation of six morphological parameters: Degree of keratinization, nuclear pleomorphism, mitosis, pattern of invasion, stage of invasion and lymphoplasmacytic infiltrate. Each parameter was given a score from 1 to 4 according to its extent (Table 1). Total points scored by each tumor was calculated by adding the points of each parameter and graded from I to IV [7,8]. One representative section from tumor block was subjected to immunohistochemical staining for Bcl-2. Pattern of Bcl-2 expression may be nuclear, cytoplasmic, membranous or any combination of these [9]. The Bcl-2 score was done according to the fraction of stained tumor cells. The score of bcl-2 was evaluated as negative (0) when no positive cell were observed within the tumour, 1+ when up to 30% of the tumour cells were positive, 2+ when 31%–70% of the tumour cells were positive and 3+ when 71–100% of tumour cells were positive [10]. A descriptive study was carried out for all the variables included in the study. The whole data was entered in Microsoft excel master sheet and analyzed using SPSSv20 software. The results obtained were interpreted and descriptive statistics were applied wherever appropriate. Where the data was qualitative, chi square test was used to assess the association between these parameters. A value of p < 0.05 was taken as significant and <0.01 as highly significant whereas p value > 0.05 was taken as non-significant. All the clinicopathological parameters and six morphological parameters of Anneroth's grading were recorded [7]. A descriptive study was carried out to compare the results of bcl-2 expression in various grades of squamous cell carcinoma of oropharynx. The work has been reported in line with the STROCSS criteria [11].

**Table 1:** ANNEROTH’S multifactorial grading system.

Morphological Parameter	Histological score			
	1	2	3	4
1. Keratinization Degree	>50% of cells keratinized	20–50% of cells keratinized	5–20% of cells keratinized	0–5% of cells keratinized
2. Nuclear pleomorphism 3. Number of mitosis/hpf	Little nuclear pleomorphism 0–1	Moderately abundant nuclear pleomorphism. 2–3	Abundant nuclear pleomorphism. 4–5	Extreme nuclear pleomorphism. >5
4. Pattern of Invasion 5. Stage of invasion	Pushing, well-delineated infiltrating borders. Carcinoma in situ and/or questionable invasion.	Infiltrating, solid cords, bands and/or strands. Distinct invasion but involving lamina propria only.	Small groups or cords of infiltrating cells. Invasion below lamina propria adjacent to muscles, salivary gland tissues, and periosteum.	Marked and widespread cellular dissemination in small groups and/or in single cells. Extensive and deep invasion replacing most of the stromal tissue and infiltrating jaw bone
6. Lymphoplasmacytic Marked Infiltrate		Moderate	Slight	None
**Grade of tumor**				**Total score**
Grade I				5–10
Grade II				11–15
Grade III				16–20
Grade IV				20+

## 3 Results

In the present study, a total of 75 cases of oropharyngeal SCC constituted the study group, during the period of 2019–20, with the age of patients ranging from 34 to 85 years. Mean age at presentation was 56.63 years. M: F Ratio in our study was 24:1.

The most common presentation was dysphagia and throat pain (84%) followed by neck swelling (6.7%). Out of total 75 cases, 66(88%) were smokers, 29(39%) consumed alcohol and 37(49.3%) gave history of tobacco chewing. The most common location of tumor was base of tongue (61.3%) followed by tonsil (24%). Lymph nodes were enlarged in 39 (52%) cases and could not be palpated on clinical examination or enlarged on USG in 36 (48%) cases.

**Fig. 1:**
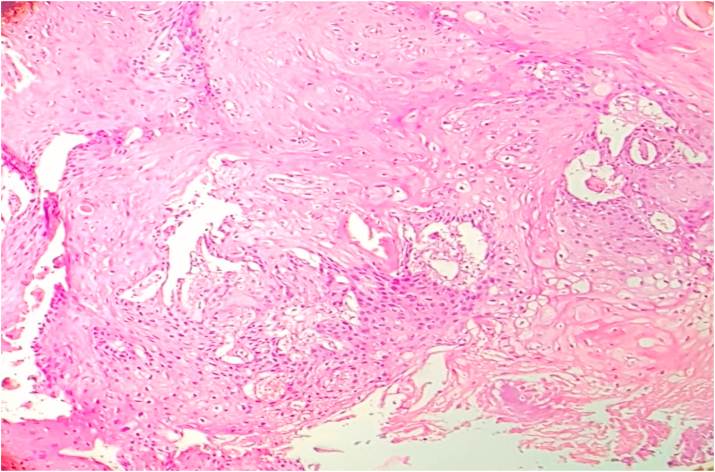
Biopsy base of tongue: Well Differentiated Squamous Cell Carcinoma (H&E, 40X).

**Fig. 2:**
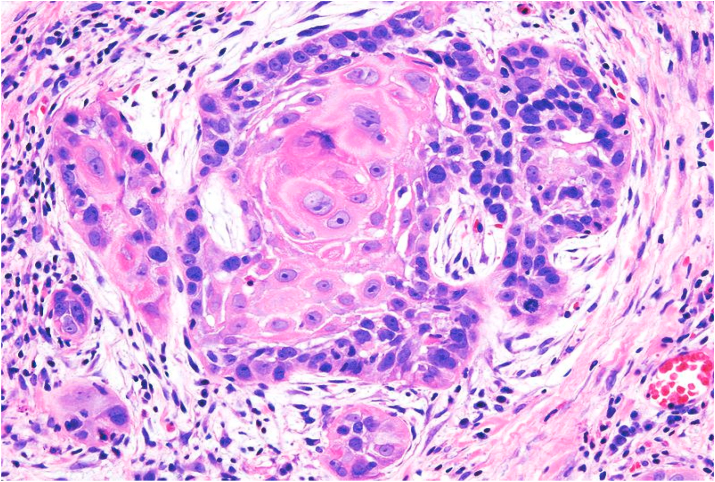
Biopsy base of tongue: Moderately Differentiated Squamous Cell Carcinoma (H&E, 200X).

**Fig. 3:**
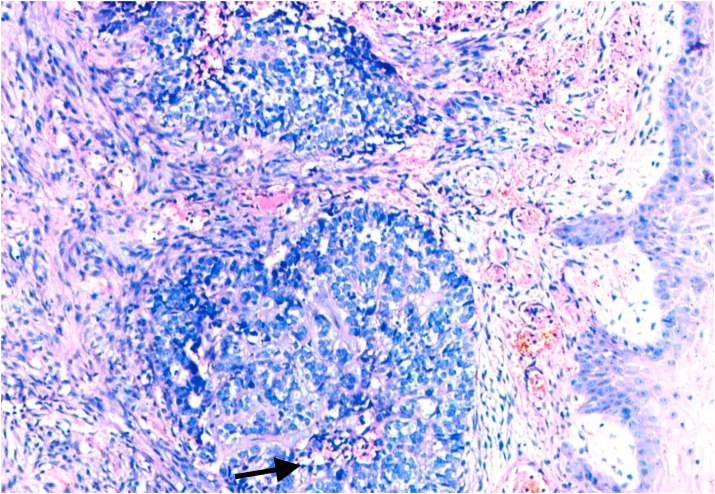
Biopsy base of tongue: Poorly Differentiated Squamous Cell Carcinoma; neutrophilic infiltration [arrow] (H&E, 100X).

The tumors were graded according to WHO grading system into well, moderately and poorly differentiated. 2.7% cases were well differentiated, 89.3% were moderately differentiated and 8% were poorly differentiated. Tumors were graded according to Anneroth's multifactorial grading system for squamous cell carcinoma of oral region taking into consideration six morphological parameters of Anneroth's grading. Out of 75 cases, 2 (2.7%) cases scored 1, 17 (22.7%) cases scored 2 and 37.3% cases each scored 3 and 4 for degree of keratinazation. For nuclear pleomorphism 13.3% cases scored 1, 70.7% cases scored 2, 14.7% cases scored 3, and 1.3% cases scored 4. For mitotic count in Anneroth's multifactorial grading system 12 (16%) cases scored 1, 38 (50.7%) cases scored 2, 20 (26.7%) scored 3 and 5 (6.6%) cases scored 4. For pattern of invasion a score of 1 was given for pushing, well delineated infiltrating borders, score of 2 was given for infiltrating solid cords, bands and/or strands, score of 3 was given for small groups or cords of infiltrating cells and a score of 4 was given for marked and widespread cellular dissemination in small groups and/or in single cells. Out of 75 case, maximum (n:43;57.3%) cases scored 3, followed by (n:21;28%) scored 2 and (n:11;14.7%) cases scored 4 and none scored score 1. For stage of invasion a score of 1 was given for carcinoma in situ and/or questionable invasion, score of 2 was given for distinct invasion but involving lamina propria only, score of 3 was given for invasion below lamina propria adjacent to muscles, salivary gland and periosteum and a score of 4 was given for extensive and deep invasion replacing most of stromal tissue and infiltrating jaw bone. Out of 75 cases, 56 (74.7%) cases scored 3 and 19 (25.3%) cases scored 2 and none scored score 1 and 4. Score 1 was not scored by any case as no case in early stage (dysplasia/carcinoma in situ) were detected in the biopsy submitted for analysis and all cases presented at an invasive stage only. For lymphoplasmacytic infiltrate 6.7% cases scored 1, 68% cases scored 2, 24% scored 3 and 1.3% cases scored 4. In Anneroth's multifactorial grading system over all grading of tumor was done by adding score of all 6 morphological parameters. Out of 75 cases, 3(4%) cases in grade I (Fig. 1), 33(44%) in grade II (Fig. 2), 39(52%) cases in grade III (Fig. 3), and no case was in grade IV.

The correlation between Anneroth's grading system and WHO grade was found to be statistically significant, χ ^2^ [degree of freedom (d.f.) 6, n = 75] = 9.52, p value = 0.049 while correlation between WHO grade with lymph node status was found to be statistically non significant, χ ^2^ (d.f. 2, n = 75) = 2.79, p value = 0.25. On the other hand, correlation between Anneroth's grade with lymph node status was found to be statistically highly significant, χ ^2^(d.f. 3, n = 75) = 12.765, p value = 0.0017 (p value < 0.01).

**Fig. 4:**
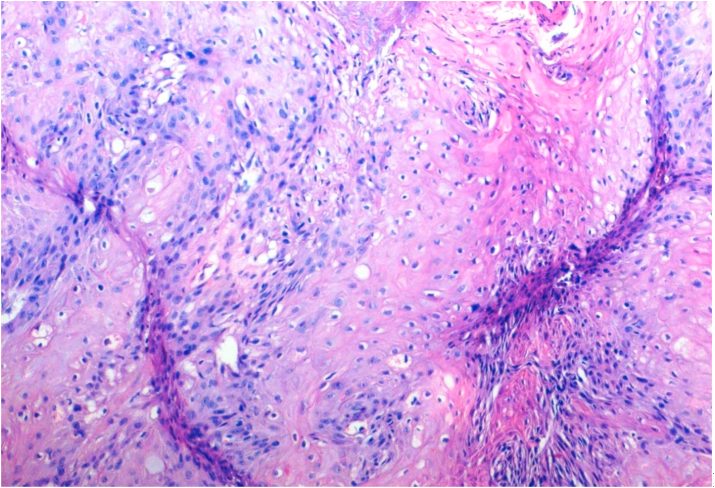
Biopsy base of tongue: Squamous Cell Carcinoma; Anneroth's Grade I (H&E, 100X).

**Fig. 5:**
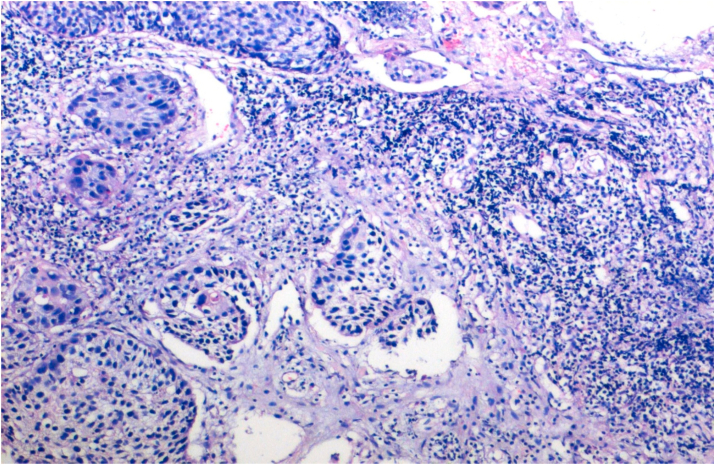
Biopsy base of tongue: Squamous Cell Carcinoma; Anneroth's Grdae II (H&E, 200X).

**Fig. 6:**
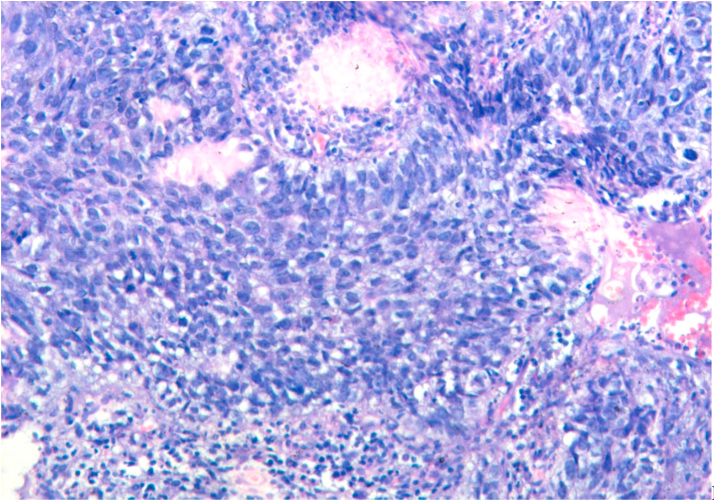
Biopsy base of tongue: Squamous Cell Carcinoma; Anneroth's Grdae III (H&E, 200X).

**Fig. 7:**
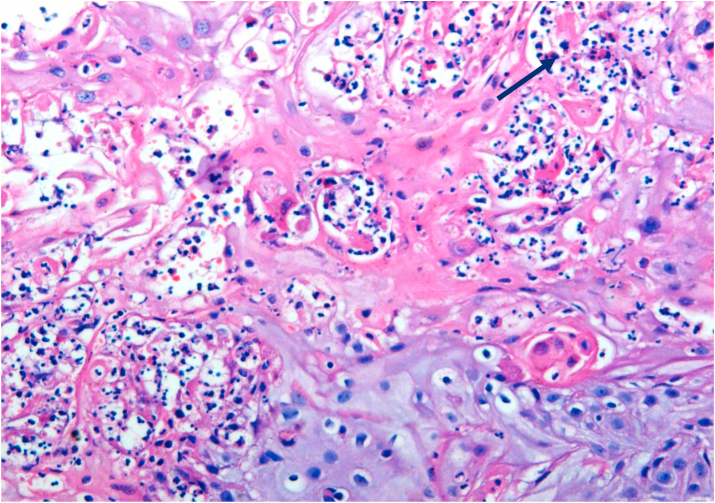
Biopsy valeculla: SCC; prominent eosinophilic infiltration [arrow] (H&E, 200X).

Bcl-2 scoring was done in the tumor parenchyma. Out of 75 case, 37(49.3%) cases scored 1+, 15(20%) scored each 2+ and 3+, and 8(10.7%) scored 0(negative) (Fig. 4, Fig. 5, Fig. 6, Fig. 7). 89.3% cases were positive for bcl-2 expression by IHC. All these 67 cases showed cytoplasmic staining; of which 15 (20%) showed nuclear positivity and 16 (21.3%) showed strong membranous positivity. All 3 patterns (nuclear, cytoplasmic and membranous) of staining were shown by 8 cases. The correlation between WHO grade with bcl-2 expression was found to be statistically non significant, χ ^2^(d.f. 6, n = 75) = 8.006, p value = 0.237 and correlation between Anneroth's grade with bcl-2 expression was also found to be statistically non significant, χ ^2^ (d.f. 9, n = 75) = 2.62, p value = 0.85 (Table 2). The correlation between bcl-2 score and lymph node status is found to be statistically non significant, χ ^2^ (d.f. 3, n = 75) = 2.8, p value = 0.42 (Table 3). The observations then made in various clinical and pathological parameters in cases of oropharyngeal squamous cell carcinoma were critically and statistically analyzed and compared with observation of different authors.

**Fig. 8:**
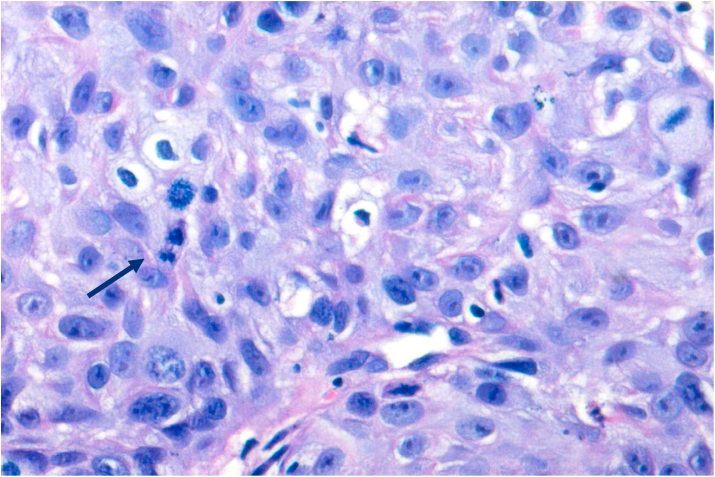
Biopsy soft plate: SCC; prominent mitotic activity [arrow] (H&E, 400X).

**Fig. 9:**
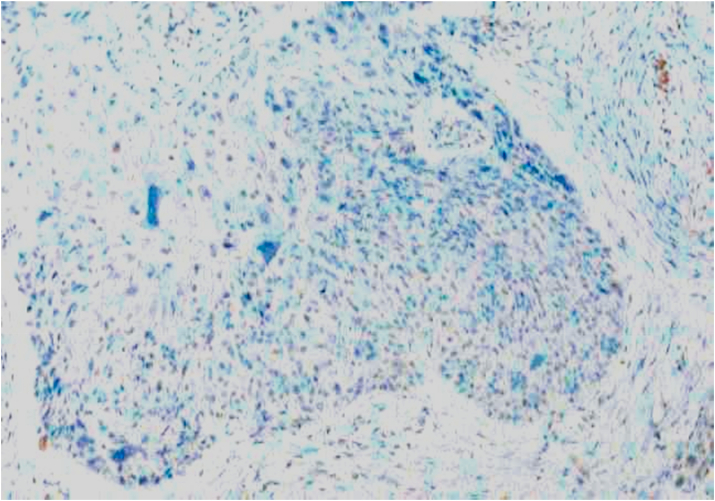
Biopsy base of tongue: MDSCC; IH C staining for Bcl-2: Negative (IHC, 100X).

**Fig. 10:**
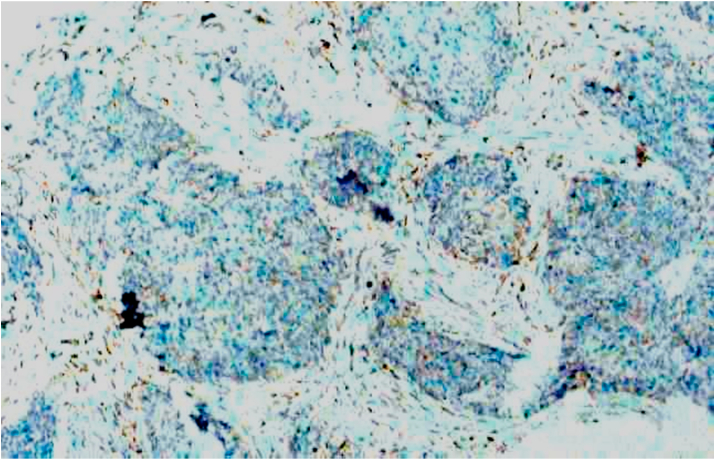
Tonsillar biopsy: MDSCC; IHC staining for Bcl-2: Positive [score: 1+] (IHC, 100X).

**Fig. 11:**
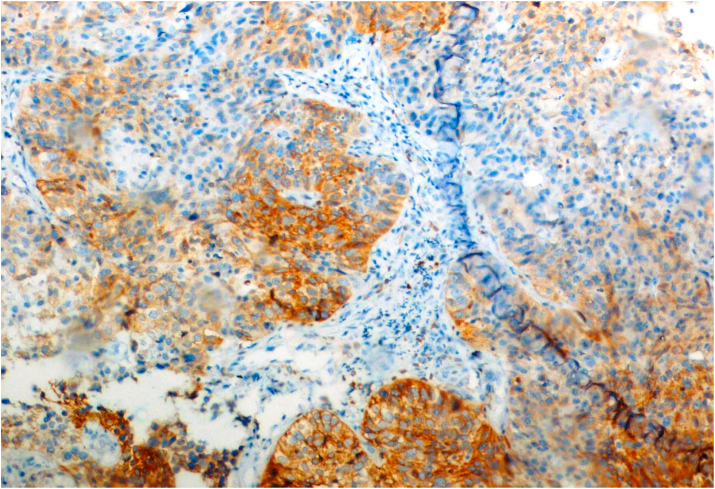
Tonsillar biopsy: MDSCC; IHC staining for Bcl-2: Positive [score: 2+] (IHC, 200X).

**Fig. 12:**
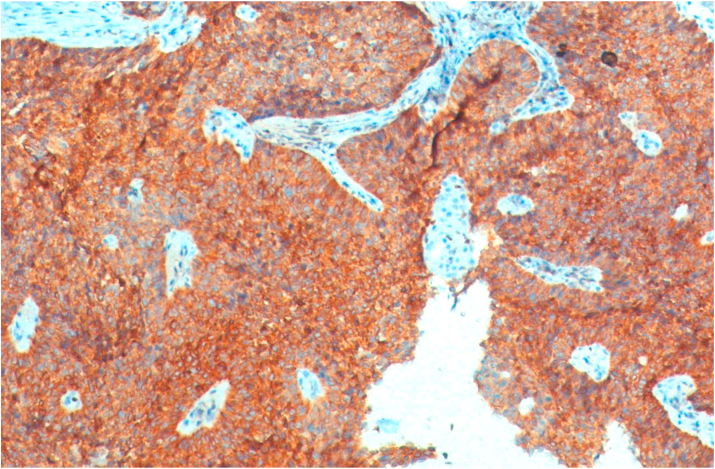
Biopsy valeculla: MDSCC; IHC staining for Bcl-2: Positive [score: 3+] (IHC, 100X).

**Table 2:** CORRELATION of ANNEROTH’S grade with BCL-2 expression (n = 75).

ANNEROTH GRADE	BCL-2 EXPRESSION				P Value
	**NEGATIVE**	**1+**	**2+**	**3+**	
**GRADE I (n = 3)**	**1(33.3%)**	**1(33.3%)**	**1(33.3%)**	**0**	**Chi square** **= 2.62,** **P value** **= 0.85**
**GRADE II(n = 33)**	**4(12.1%)**	**18(54.5%)**	**6(18.2%)**	**5(15.2%)**	
**GRADE III(n = 39)**	**3(7.7%)**	**18(46.2%)**	**8(20.5%)**	**10(25.6%)**	
**GRADE IV(n = 0)**	**-**	**-**	**-**	**-**	
**TOTAL(n = 75)**	**8(10.7%)**	**37(49.3%)**	**15(20%)**	**15(20%)**

**Table 3:** CORRELATION between BCL-2 score and lymph node status.

LYMOH NODE STATUS	BCL-2 SCORE				P VALUE
	NEGATIVE	1+	2+	3+	
**Enlarged(n:39;52%)**	**5(62.5%)**	**16(43.2%)**	**8(53.3%)**	**10(66.7%)**	**Chi Square = 2.8** **P value = 0.42**
**Not Enlarged(n:36;48%)**	**3 (37.5%)**	**21(56.8%)**	**7(46.7%)**	**5(33.3%)**	
**TOTAL(N = 75)**	**8**	**37**	**15**	**15**	

## 4 Discussion

Squamous cell carcinoma (SCC) comprises 90% of orophayngeal malignancies [1]. Carcinomas of oropharynx are characterized by genetic heterogeneity, exhibiting a wide variety of clinical presentations and of disease aggressiveness in different patients and ethnic populations**.** Despite aggressive and multidisciplinary treatment approaches, including preoperative or postoperative chemotherapy and/or radiotherapy with reconstructive surgery, there has been no significant improvement in 5 year survival over the past 20 years. Treatment failure still occurs in the form of loco-regional recurrence, distant metastasis, and/or second primary tumors. Currently treatment strategies rely on clinical, radiologic and histopathologic parameters to determine the stage of the disease. It is widely accepted that presence of lymph node metastasis is the most adverse independent prognostic factor in head and neck squamous cell carcinoma (HNSCC) [12]. Although the grade of the tumor is not included in the staging of the tumor, it should be recorded. Grade is also a powerful predictor of distant metastasis and an important factor in determining the clinical and pathologic neck staging. It helps to identify patients at high risk of distant metastasis for whom an efficient systemic treatment is mandatory [13]. Although IHC-based assays do not provide as much biological insight into tumor biology as gene-based ones do, the former are being increasingly used as a surrogate for molecular gene profiling since they allow classification of tumors at affordable costs and in the absence of fresh tissue specimens [12].

Present study was conducted with the aim to study the bcl-2 expression in squamous cell carcinoma of oropharynx and to correlate expression of bcl-2 with tumor grade.

The age and sex distribution of present as well as previous studies indicate that the incidence of oropharyngeal carcinoma is higher in older age group and in males. This can be attributed to tobacco chewing or smoking and alcohol consumption being more common amongst males in our part of the world which play an important role in the etiopathogenesis of SCC of oropharynx [2,3]. The association between smoking and specific genetic alterations in patients with HNSCC has been well documented in the literature and also correlates with our above observation [14]. Though tongue was the most common site of involvement in most of the studies, the difference in percentage could be because of different geographical location and prevalence of risk factors.

Histologic grade is a strong and independent factor associated with distant metastasis in oropharyngeal carcinomas. Thus it adds important information to clinical and pathologic staging. It helps to identify patients at high risk for distant metastasis for whom an efficient systemic treatment is mandatory [15].

AJCC TNM staging is the most commonly used method in clinical practice nowadays. However, this system does not provide any information on the biological characteristics and thus the aggressive clinical behaviour of tumor. Thus in our study, tumors were graded according to Anneroth's multifactorial grading system for squamous cell carcinoma of oral region. The dissimilarity in frequency of different grades with other studies can be attributed to a wide interobserver variability as the grading is on the basis of six different morphological parameters is subjective.

While the role of bcl-2 immunoexpression has been widely investigated, staining intensity and distribution cut offs are not universally standardized and different authors have used different criteria's for the same. In our study, bcl-2 scoring was done taking into consideration the number (percentage) of positive cells in the tumor parenchyma as described by Xie et al. In study done by Xie et al., bcl-2 immunoreactivity was found in 83(97%) out of 85 cases [10]. Sulkowska et al. observed positive IHC staining for bcl-2 protein in more than 10% of tumor cells in 27% of oral cancer cases [16]. Ravi et al. observed bcl-2 positivity in 87% of cases [17]. Sudha et al., observed bcl-2 immunoreactivity in 26(86.7%) out of 30 cases [18]. Suri et al., observed bcl-2 immunoreactivity in all 32(100%) cases [19]. Our study was in disconcordance with the study of Saikrishna et al. which showed bcl-2 positivity in 12(18%) out of total 67 cases [20]. This may be due to authors choosing semi quantitative labeling index by counting 1000 cells in 8 randomly chosen fields' on10× magnification [5].

In our study the correlation between Anneroth's grading system and WHO grade was found to be statistically significant, χ ^2^(d.f. 6, n = 75) = 9.52, p value = 0.049 (p value less than 0.05). In present study well differentiated tumors were in Anneroth's grade I and poorly differentiated tumor were in Anneroth's grade III. Moderately differentiated tumors were equally distributed in Anneroth's grade II and III. Thus Anneroth's grading system subdivided MDSCC into two main categories (grade II and III) but its long term effect on prognostic factors and survival needs to be evaluated further.

In our study the correlation of WHO grade with bcl-2 expression was found to be statistically non significant, χ ^2^(d.f. 6, n = 75) = 8.006, p value = 0.24 and the correlation between Anneroth's grade and bcl-2 expression was also found to be statistically non significant, χ ^2^(d.f. 9, n = 75) = 2.62, p value = 0.85.

In the study conducted by Lehnerdt et al. the correlation of WHO grade with bcl-2 expression was found to be statistically non significant, p value = 0.393 [21]. In the study done by Thongsuksai et al. the correlation of WHO grade with bcl-2 expression was found to be statistically non significant, p value = 0.07 [22]. However in the study of Drenning et al. the correlation of WHO grade with bcl-2 expression was found to be statistically significant, p value = 0.045 [23]. In the study of Sulkowska et al. the correlation of WHO grade with bcl-2 expression was found to be statistically highly significant, p value = <0.005 [16]. In the study of Helal et al. the correlation of WHO grade with bcl-2 expression was statistically significant, p value = <0.05 [9]. In the study conducted by Suri C to compare the number of cells expressing bcl-2 in different grades of oral SCC, the p value was found to be very highly significant (p = 0.001) [19]. Similarly in the study done by Saikrishna et al. showed sequential increase in bcl-2 positivity from 16 to 25% when tumor grade changes from well differentiated to moderate to poorly differentiated SCC [5]. There are no studies available to compare our findings of correlation of bcl-2 expression with Anneroth's grade.

There are different studies revealing different levels of significance of bcl-2 expression when analyzed in context of grade of tumor from non significant to highly significant. In the present study bcl-2 expression did not show any significant association with grade of tumor. This may be due to small number of cases of well and poorly differentiated tumors being evaluated.

The expression of bcl-2 rises with increasing grade of tumor. Poorly differentiated SCC show higher Bcl-2 positivity followed by moderately differentiated. Well differentiated SCC show the least Bcl-2 positivity. Patients whose tumor demonstrate bcl-2 positivity even with locoregionally advanced disease, appear to have a high likelihood of cure with aggressive combined modality therapy and may be treated successfully with less toxic therapy. Bcl-2 status is closely associated to the success of chemoradiation treatment of oropharyngeal SCC. Patients of oropharyngeal SCC with pretreatment staining for bcl-2 had much shorter disease free survival. Elevated bcl-2 expression in primary tumor is associated with significantly increased risk of recurrence. It helps to identify patients at high risk for unfavourable outcome [21].

In our study the correlation between WHO grade with lymph node status was found to be statistically non significant, χ ^2^(d.f. 2, n = 75) = 2.79, p value = 0.25 whereas the correlation between Anneroth's grade with lymph node status was found to be statistically highly significant, χ ^2^(d.f. 3, n = 75) = 12.765, p value = 0.0017(p value < 0.01). The correlation between bcl-2 score and lymph node status was found to be statistically non significant, χ ^2^(d.f. 3, n = 75) = 2.8, p value = 0.42. The reason for poor correlation with lymph node metastasis or prognosis, is the relative heterogeneity of the cell population present in tumor cells [20].

The difference in the study of doshi et al. for association of lymph node metastasis with Anneroth's grading system may be attributed to slightly different parameter observed by them for overall grading of tumor. In their study grade I was given for total score of 6–12, grade II for a score of 13–18 and grade III for total score of 19–24.

In the study conducted by Drenning et al. there was no statistically significant correlation between lymph node metastasis and bcl-2 positivity (p value = 0.09) [23]. Similarly in the study conducted by Thongsukai et al. there was no statistically significant correlation between lymph node metastasis and bcl-2 positivity (p value = 0.60) [22]. The study done by Lehnerdt et al. also did not show any correlation between lymph node metastasis/TNM staging and bcl-2 positivity (p value = 0.78). All these studies are in concordance with our study [21].

In present study although there was no correlation between expression of bcl-2 and the grade of the tumor but overall 89% of the cases revealed bcl-2 expression in tumor cells. For conclusive statement regarding correlation of bcl-2 expression with grade of the tumor requires more and larger studies. However the corollary finding was significant role of Anneroth's grading system in predicting the stage of tumor [24].

## 5 Conclusion

There was significant correlation between Anneroth's grading system with WHO grading system in SCC of oropharynx. WHO grading system did not predict the stage of tumor as there was no significant correlation between tumor differentiation and lymph node metastasis. But Anneroth's grading system was found to be more relevant in predicting the stage of tumor. Bcl-2 expression did not correlate with the grading of tumor (both WHO and Anneroth's). Larger studies and correlation with genetic analyasis are required to reach conclusive significance of bcl-2 expression in grading, staging and outcome in oropharyngeal SCC.

## Source of funding

Nil.

## Data availaibility statement

Data will be available to all the readers as per journal's rules.

## Provenance and peer review

Not commissioned, externally peer-reviewed.

## Authors contribution

1. Dr. Namita Bhutani: written the manuscript
2. Dr. Pooja poswal: data collection
3. Dr. Shilpi Moga: data collection
4. Dr. Sunil Arora: assisted in framing manuscript

## Ethical approval

Yes approval was given by ethical commitee of sgt medical college, hospital and research institute.

Reference no: FMHS/MD/23112020 DATED: 23.11.2020.

## Guarantor

Dr. Namita Bhutani.

## Declaration of competing interest

We do not have any conflict of interest.
